# Marine iguanas have lower metabolic rates during El Niño

**DOI:** 10.1242/jeb.250907

**Published:** 2025-09-05

**Authors:** Shahar Dubiner, Juan Pablo Muñoz-Pérez, Gregory A. Lewbart, Kenneth J. Lohmann, Maximilian Hirschfeld, Daniela Alarcón-Ruales, Thara Carolina Cango Rivadeneira, Andrea Loyola, Shai Meiri, Eran Levin

**Affiliations:** ^1^ School of Zoology, Faculty of Life Sciences, Tel Aviv University, Tel Aviv 6997801, Israel; ^2^ Universidad San Francisco de Quito (USFQ) & UNC-Chapel Hill Galápagos Science Center (GSC) Av. Alsacio Northia, Puerto Baquerizo Moreno, Isla San Cristóbal, Galápagos, Ecuador; ^3^ School of Science, Technology and Engineering, University of the Sunshine Coast UniSC, Hervey Bay, Australia; ^4^ North Carolina State University College of Veterinary Medicine, 1060 William Moore Drive, Raleigh, NC 27607, USA; ^5^ Department of Biology, University of North Carolina at Chapel Hill, Chapel Hill, NC 27599, USA; ^6^ James Cook University, Townsville, QLD, Australia; ^7^ Dirección Parque Nacional Galápagos, Puerto Ayora, Isla Santa Cruz, Ecuador; ^8^ The Steinhardt Museum of Natural History, Tel Aviv University, 6997801, Israel

**Keywords:** Algae, *Amblyrhynchus cristatus*, Body condition, Food shortage, Ocean warming, Reptile

## Abstract

The Galápagos marine iguana (*Amblyrhynchus cristatus*), the world's only marine lizard, feeds predominantly on algae. Owing to warming waters and reduced upwelling, algal abundance is reduced during El Niño events, causing high iguana mortality. During such periods, adult iguanas may shrink in size, a compelling phenomenon that has been suggested as an adaptation to reduce energetic needs. However, shifts in energy consumption have never been tested directly. We measured the body condition and metabolic rates of marine iguanas during an El Niño year and the subsequent neutral year. During El Niño, body mass relative to length was 17% lower, girth relative to length was 12% lower, and resting metabolic rates were 20% lower. This supports the hypothesis that marine iguanas partly offset the adverse effect of El Niño by an active response aimed at reducing their energy consumption, complementary to the energy-saving effect of body size reduction. Future ocean warming could force this endemic species to resort to such strategies increasingly often, and will likely exacerbate the already-high mortality rates caused by these events.

## Teaser

**Highlighted Article:** Measurements of body condition and metabolic rates of marine iguanas during an El Niño year showed that metabolic rates are 20% lower, offsetting the adverse effects of El Niño by reducing energy demands.

## INTRODUCTION

**Fig. 1.**
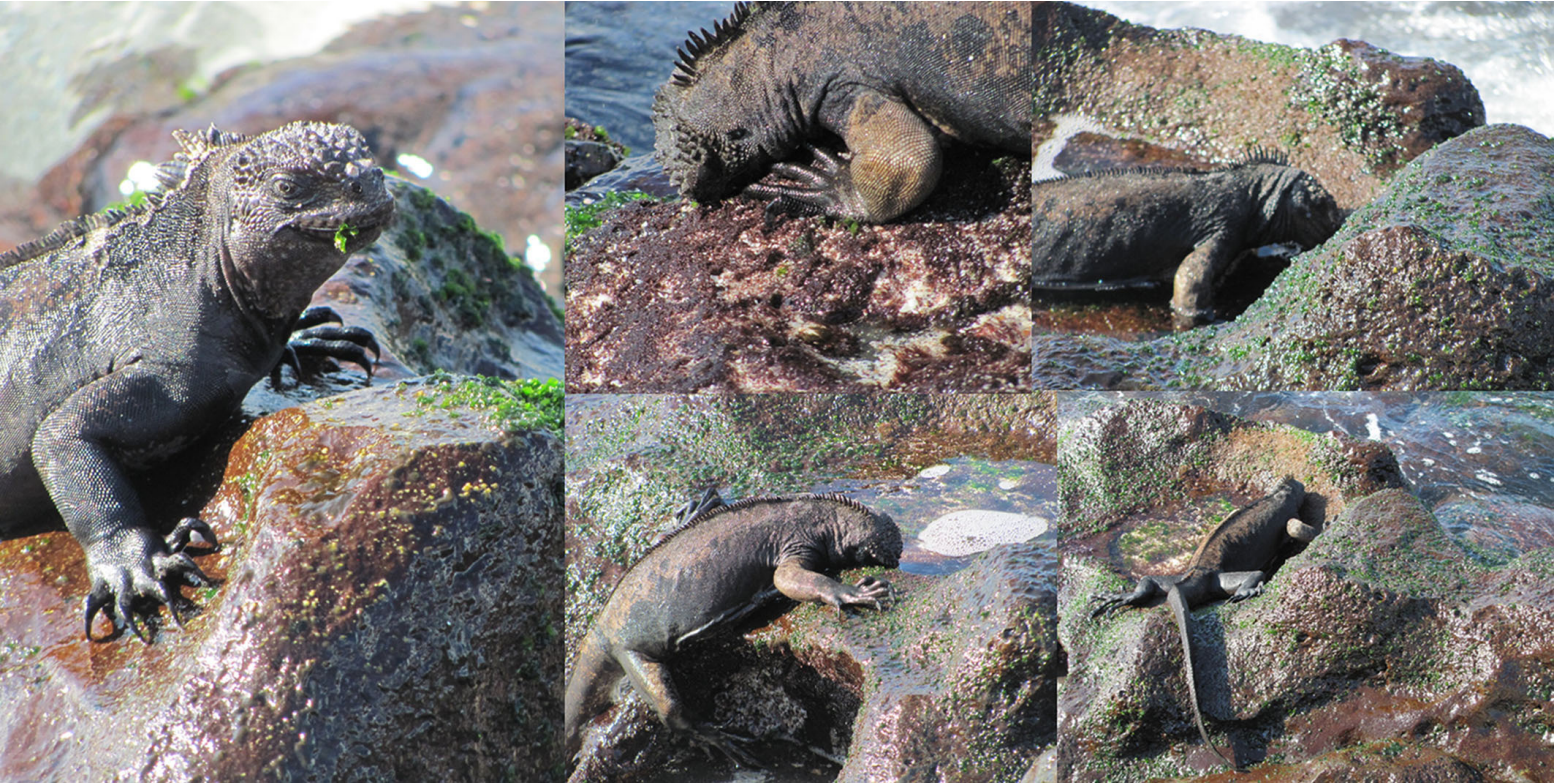
**A marine iguana grazing on algae on the intertidal rocks in Playa Baquerizo, San Cristóbal Island.** Photos by Shahar Dubiner.

The Galápagos marine iguana (*Amblyrhynchus cristatus*) is unique among the world's ∼7850 species of lizards, in being the only one that forages in the sea (Wikelski, 2005). It feeds primarily on various green and red algae (namely *Centroceras*, *Gelidium*, *Spermothamnium* and *Ulva*; Vitousek et al., 2007). Large adults can dive to below 30 m, where algae are most abundant (Wikelski and Nelson, 2004), whereas smaller, younger individuals mostly forage in the shallows and on rocky shores during low tide (Vitousek et al., 2007; Fig. 1). Food availability in the Galápagos can dramatically change from year to year, due to the El Niño–Southern Oscillation (ENSO), a highly unpredictable yet frequent phenomenon in which sea surface temperatures (SSTs) greatly increase near the islands for many months. Following El Niño events, the combination of warming mean temperatures (commonly by 0.5–2.5°C; NOAA, 2024a) and reduced upwelling of nutrient-rich water induces massive dieback of the algae species that iguanas eat (Laurie, 1989; Rubenstein and Wikelski, 2003). These events result in high mortality from starvation; in extreme cases, more than half the individuals across the archipelago may perish (Laurie, 1990), though different subspecies are affected to different degrees. In the most susceptible populations, mortality can reach 90% following El Niño (Romero and Wikelski, 2001; Wikelski and Wrege, 2000).

The selective pressure imposed by these events gave rise to a curious response in marine iguanas to the sudden and prolonged food shortages: surviving individuals were observed to shrink in body length by over 20% following El Niño events (Wikelski and Thom, 2000). Changes in body size are known to occur in many reptile species following periods of food shortage, through plasticity of visceral organ size (Dubiner et al., 2024; Secor and Lignot, 2010). However, *A. cristatus* is unique in that it accomplishes shrinking by resorption of body tissues – not only soft tissues but cartilage and bone too (Vitousek et al., 2007). This reduces the iguana's energetic expenditure to a more efficient level relative to their foraging capacity (Wikelski and Thom, 2000). In years when algae rebound, the iguanas grow larger once more (Wikelski and Romero, 2003). It has been suggested that shrinking is a mechanism for energetic saving, because smaller iguanas have overall lower metabolic needs if a constant size-dependent energy consumption is assumed (Wikelski and Romero, 2003). However, this has a negative impact on other size-related traits, namely breeding (Vitousek et al., 2007). Energy consumption could potentially be reduced via complementary pathways, e.g. by selecting lower body temperatures (Christian et al., 1983; M. Wikelski, pers. comm.) or even lowering the metabolic rate directly. The latter is consistent with the observation of slower heart rates (a proxy for metabolic rates; Green, 2011) during the three most recent El Niño events (Dubiner et al., 2025). We hypothesized that the lower metabolic activity may help the iguanas cope with food shortage by reducing their energetic needs. Past studies have shown that squamate metabolic rates are often lower in regions with low food availability (Dubiner et al., 2023; Giacometti et al., 2022), and during periods of reduced food intake, such as dormancy (Christian et al., 1999; Dubiner et al., 2023, 2024; Milsom et al., 2008; Tsuji, 1988). Elucidating metabolic shifts in marine iguanas could illuminate similar phenomena in other ectotherms. El Niño is an episodic event that results in food shortages, to which there are seasonal parallels in many regions, such as winter dormancy in response to the cold (Dubiner et al., 2023; Tsuji, 1988). In the tropics (including the Galápagos; Christian et al., 1983), dry seasons create the challenge of low food availability even at favorable environmental temperatures. Known responses to these conditions include metabolic depression (i.e. reduction of metabolic activity), thermoregulation to lower body temperatures, and reduced activity (Christian et al., 2024).

In this study, we measured iguana metabolic rates to investigate whether energetic expenditure is reduced during El Niño years. Despite extensive research showing reduced food intake (Vitousek et al., 2007; Wikelski, 2005; Wikelski and Romero, 2003; Wikelski et al., 1993), the effects of El Niño events on iguana metabolism have never been directly tested. We measured subadults and juveniles, the life stages that are the most vulnerable because they are exposed to the most drastic shifts in food availability (Laurie and Brown, 1990a,b), owing to high competition and restriction to shallow/intertidal waters. Furthermore, small individuals tend not to shrink as much as adults (Wikelski and Thom, 2000), perhaps because shrinkage is a result of optimizing energy requirements relative to foraging abilities (Wikelski and Romero, 2003; Wikelski et al. 1997); thus, the amount of energy that small iguanas can save by shrinking is smaller. Therefore, metabolism can provide insights into alternative or complementary energy-saving solutions that have arisen in response to the pressures posed by El Niño.

## MATERIALS AND METHODS

**Fig. 2.**
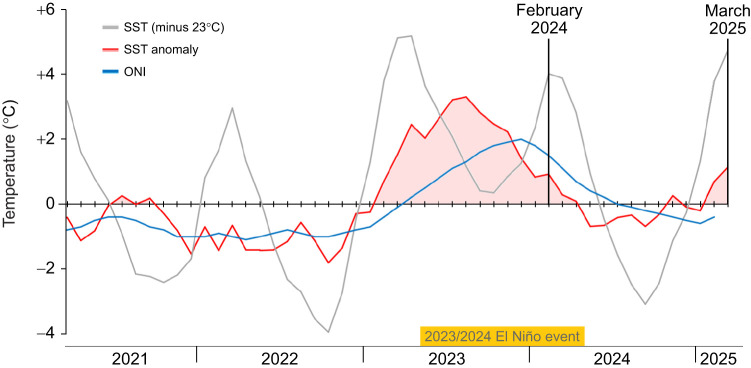
**Climatic conditions in the 4** **years prior to and during our study (two periods of measurements in mid-February 2024 and mid-March 2025).** Local sea surface temperatures (SST, in grey; zero is set at 23°C for better visualization) and their anomalies (in red) are given as monthly means for the Niño 1+2 region, which contains the island of San Cristóbal. The Oceanic Niño Index (ONI, in blue) is the 3-month mean SST anomaly in the east-central tropical Pacific; El Niño is officially defined as when ONI>0.5 for five or more consecutive months. The 2023/2024 event lasted from May to April, so the first set of measurements was preceded by a year of El Niño conditions, whereas the second was preceded by neutral conditions. Tick marks are a month apart.

We measured marine iguanas (*Amblyrhynchus cristatus* Bell 1825) during two periods: 11–14 February 2024 (*n*=47) and 11–18 March 2025 (*n*=50). The first period followed an El Niño year (the 2023/2024 event) and the other followed an ENSO-neutral year (2024/2025). We confirmed El Niño conditions using data from the National Oceanic and Atmospheric Administration (NOAA) on local SSTs and SST anomalies (NOAA, 2024b; values for the Niño 1+2 region, which contains the island of San Cristóbal), as well as the Oceanic Niño Index (ONI; units in °C), i.e. the 3-month mean SST anomaly in the Niño 3.4 region (east-central tropical Pacific; NOAA, 2024a). An El Niño is officially defined as when ONI>0.5 for five or more consecutive months. February 2024, when the first set of measurements was conducted, had a mean local SST (Niño 1+2 region) of 27.0°C, equivalent to an SST anomaly of +0.9°C. It was preceded by 12 consecutive months of SST anomalies greater than +0.7°C (averaging +2.1°C and peaking at +3.3°C), and mean ONI of +1.04, indicating strongly ENSO-positive conditions (Fig. 2). March 2025, when the second set of measurements was conducted, had a mean local SST (Niño 1+2 region) of 27.8°C, equivalent to an SST anomaly of +1.2°C. These conditions are similar to the first set, but in stark contrast, ONI during the second set was −0.4 and was preceded by 12 consecutive months of SST anomalies averaging −0.1°C, and mean ONI of −0.008°C, indicating ENSO-neutral conditions (Fig. 2).

We caught 87 marine iguanas by hand, on the beaches at three sites near Puerto Baquerizo Moreno on the island of San Cristóbal (La Lobería: 0.923°S, 89.618°W; Punta Carola: 0.896°S, 89.612°W; and near Playa Mann: 0.896°S, 89.609°W). Upon capture, we recorded standard measurements: body mass (using a digital spring scale), snout–vent length (SVL) and body girth (thorax circumference at the axillary region; Labocha et al., 2014). Of these, we proceeded to measure the resting metabolic rate (RMR) of 38 juveniles/subadults (19 each year). We focused on this age range because they are exposed to the most drastic shifts in food availability yet are not as prone to shrink as older individuals; in addition, they were the best fit for the dimensions of our incubator and metabolic chamber. The experiment was conducted as part of a wildlife health research program permitted by the Galápagos National Park Service (permits PC-04-23 and PC-36-24 to Gregory A. Lewbart) and approved by Universidad San Francisco de Quito (USFQ), North Carolina State University (NC State) IACUC ID 8-009-O, ethics and animal handling protocols. All handling and sampling procedures adhered to standard protocols for vertebrate and veterinary practices.

RMR was estimated through oxygen consumption, which was measured on-location using open-flow respirometry with an FMS Field Metabolic System (Sable Systems International, North Las Vegas, NV, USA). Flow rate of atmospheric air was set by the internal FMS pump to 500 ml min^−1^ (pull-mode) and chamber volume was 8 liters for most measurements, or in an otherwise identical 12 liter chamber for the larger individuals (more than ∼2 kg). Temperature was set to 30°C using a portable incubator (Sable Inc., USA). Power was supplied by a portable generator. Water vapor and CO_2_ could not be scrubbed from the air, owing to limitations on importing the necessary materials to the island. We therefore corrected the O_2_ dilution mathematically according to eqns 1–8 in Withers (2001). For this purpose, we also recorded CO_2_ and water vapor pressure (WVP, which indicated that chamber air was near saturation in all measurements, as is recommended for this setup; Lighton, 2008). We corrected O_2_ values for drift by taking a baseline of atmospheric air before and after the measurement. Each measurement lasted for 1–2 h until full stabilization of excurrent gas concentrations, and was recorded by computer in real-time using Expedata. If an animal was restless in the metabolic chamber for longer than a few minutes, we terminated the experiment and discarded the measurement. All iguanas were released immediately after measurements ended, which occurred within 1–2 h of capture, at the exact point of capture. Immediately prior to release, each individual was marked dorsally with dots of zinc oxide to avoid recapture. To compare the same individuals in both years, we marked most iguanas using RFID PIT tags (subcutaneously, in the left hindleg) in 2024, after measuring their RMR. Unfortunately, none of these animals were recaptured in 2025. See Table S1 for complete data.

We tested for changes in body condition using multiple linear regressions with year (2024 versus 2025, set as a factor), SVL (mm, log transformed) and study site as predictors, and either body mass (g, log transformed) or girth (mm, log transformed) as the response. We interpret higher mass and girth for a given SVL as indicative of a better body condition (Labocha et al., 2014). We performed a sensitivity analysis only including the iguanas that we measured for metabolism (*n*=36). We tested for changes in RMR using a multiple linear regression with year, body mass (g, log-transformed) and study site as predictors, and RMR (ml O_2_ g^−1^ h^−1^, log-transformed) as the response. Two juvenile individuals with outlying body mass (<600 g), measured in 2024, were removed from this analysis to avoid skewing the results (we saw no iguanas nearly as small as these in 2025, on any of the three beaches). In another sensitivity analysis we included these small individuals (*n*=38). To compare the goodness-of-fit of these models, root mean square error (RMSE) and σ were calculated using the ‘performance’ package in R.

## RESULTS

**Fig. 3.**
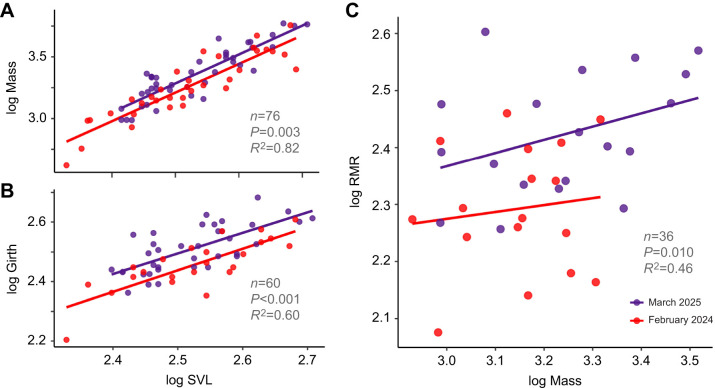
**Results of the morphological and metabolic field measurements.** (A) Body mass (g) was 17.4% lower and (B) girth (mm) was 11.8% lower in 2024 compared with 2025, indicating worse body condition following El Niño. SVL, snout–vent length (mm). (C) Resting metabolic rates (RMR; O_2_ μl h^−1^) were 20.3% lower in 2024, indicating metabolic depression following El Niño.

Body mass increased with SVL with an allometric exponent of 2.22±0.14 and was 17.4% lower, for a given length, in 2024 compared with 2025 (*n*=76, model *R*^2^=0.81, *P*<0.001; Fig. 3A). Body girth increased with SVL with an allometric exponent of 0.70±0.10 and was 11.8% lower in 2024 than in 2025 (*n*=60, model *R*^2^=0.79, *P*<0.001; Fig. 3B). A sensitivity analysis using only the 38 individuals measured for metabolism upheld these results (mass: *P*=0.036; girth: *P*=0.018).

Resting metabolic rates were lower during El Niño: mass-specific RMR decreased steeply with body mass with an allometric exponent of 0.09±0.04 and was 20.3% lower for a given mass in 2024 compared with 2025 (*n*=36, *R*^2^=0.46, RMSE=0.09; σ=0.10; *P*=0.010; Fig. 3C). Sensitivity analysis that included the body size outliers upheld these results (*n*=38, *R*^2^=0.34, RMSE=0.10, σ=0.11, *P*=0.036). Allometric slopes did not differ between years (i.e. year×mass×RMR interactions were not significant; *P*=0.557).

## DISCUSSION

Our study provides the first direct evidence that marine iguanas reduce their energy demands during El Niño. The resting metabolic rates of juvenile and subadult *A. cristatus*, when adjusted for other influencing factors, were reduced by 20% following an El Niño year and its exceptionally high sea temperatures (reaching 3.3°C above average in the cold season). Controlling for site and body size, at a constant, ecologically relevant body temperature, we show that iguanas have much slower metabolism, independent of (and thus additional to) any saving achieved by size reduction itself. Concomitantly with the lower metabolic rates, the iguanas' body condition was lower, with both mass and girth being lower in relation to body length. These findings are consistent with the interpretation that marine iguanas are physiologically adapted to modulate their energy demands in response to the reduced energy supply following the reduction in algal abundance and subsequent prolonged hunger. Although iguanas are known to reduce their energetic intake (Vitousek et al., 2007; Wikelski, 2005; Wikelski and Romero, 2003; Wikelski et al., 1993) and heart rates (Dubiner et al., 2025) during El Niño, it has not previously been investigated whether they also reduce their metabolic rates.

A reduction in metabolic rate can be achieved by allocating resources away from growth and reproduction. Growth rates account for a portion of energy allocation in juveniles (Nagy, 2000) and are highly plastic in reptiles (Meter et al., 2020); thus, switching to lower growth can save energy even in individuals too small to shrink. Wikelski and Thom (2000) reported that although juveniles maintained body size or even grew, growth rates were lower during El Niño. Alternatively, or additionally, iguanas could reduce their size of internal organs, which account for most of the body's energy consumption (Nespolo et al., 2002). Such organ-shrinking was observed in other reptiles, and was shown to be an energy-saving mechanism during periods of low food intake (Dubiner et al., 2024; Secor and Lignot, 2010). Thus, the reduction in body mass and girth (relative to length) may be due to not only loss of fat but the degradation of size-flexible tissues such as the heart, gastrointestinal tract and liver, or muscle tissue.

Differences in thermal conditions between years could also influence RMR irrespective of food availability. Even though weather conditions and SSTs were similar in both sampling campaigns (differences were in the year leading up to sampling; Fig. 2). Prolonged exposure to anomalous temperatures may influence metabolism and this effect may remain regardless of the conditions at the time of measurement. Ectotherms exposed to varied thermal regimes for prolonged periods, but tested at the same experimental temperatures, can display different metabolic rates (Giacometti and Tattersall, 2025; Patterson and Davies, 1984; Tsuji, 1988). Although air temperatures on land during El Niño are not very noticeable, the drastic change in sea temperatures (which can be very cold in ENSO-neutral years; NOAA, 2024b) may be enough to trigger acclimatization. Also, feeding status could hypothetically play a role in passively reducing metabolic rates, because fasting reptiles have lower rates than fed ones, because of a phenomenon which elevates metabolic rates following meals in reptiles (specific dynamic action, SDA; Secor, 2009). Although SDA is less significant in herbivorous, continuously feeding lizards than in other reptiles such as snakes, the difference in metabolic rates between fully fed and starved green iguanas (*Iguana iguana*) are nearly halved (Guagnoni et al., 2024). However, we think this is unlikely to be the only factor at play in our case, as our measurements were taken when conditions were beginning to return to normal, when the iguanas were often observed feeding.

Energetic expenditure can be lowered by downregulating, inhibiting and decreasing the rate of costly metabolic pathways, a process known as metabolic depression (Guppy and Withers, 1999). This could explain the reduction in RMR, with or without acclimatization, changes in body size or temperature differences. Metabolism is size- and temperature-dependent in ectotherms (Seebacher, 2005), but metabolic depression can have a large effect independent of these relationships, and is very common in reptiles. In regions where cold or dry seasons limit or prevent foraging, seasonal metabolic depression is ubiquitous (Christian et al., 2024; Dubiner et al., 2023; Tsuji, 1988). It often appears to be induced by the lack of food and not by cold temperatures (Dubiner et al., 2024), and was shown to be most efficient in warm climates and for large body sizes (Christian et al., 1999; Milsom et al., 2008). Consequently, we suggest that marine iguanas use such mechanisms to reversibly lower their energetic needs during El Niño events, until conditions become favorable again. However, such a reduction will potentially come at the expense of their ability to forage, grow and reproduce (Vitousek et al., 2007). Though a 20% metabolic depression is substantial in terms of energy conservation, it is low compared with the rates reported for hibernating and aestivating squamates (Christian et al., 1999; Dubiner et al., 2023; Tsuji, 1988). In contrast to brumating (dormant) squamates, iguanas remain physically active during algal dieback periods, and still forage, swim and walk to and from the beaches. Therefore, they cannot forsake many functions that brumating lizards do while inactive.

Our estimate of the energy saved by reduced metabolism is probably conservative, for two reasons. First, we adjusted RMR to body mass, which might have introduced a bias if reduced body condition had been solely due to loss of fat (metabolically active tissue mass would then be the same while total mass was lower). However, if this were the case it would have led to the opposite trend: higher mass-specific RMR during El Niño. Second, our measurements were taken after the sea temperatures were starting to decrease (2024) or increase (2025; Fig. 2), meaning that RMR was not necessarily at its extreme high or low, respectively. This could mask a larger underlying metabolic depression than we report. Even if such rates of metabolic depression are not enough to balance the reduced food intake on their own, they are complementary to other strategies. Adaptations such as body shrinking (Wikelski and Thom, 2000) and changes in thermoregulation (Christian et al., 1983), alongside passive effects of feeding status and acclimatization, could account for the majority of the energetic saving, with metabolic depression providing an additional 20%, which is potentially crucial to bring them over the ‘finish line’.

Assessing the adaptations to warming-induced food shortages can provide insights into animals' responses to temporary harsh conditions. It will help us understand, predict and prepare for the effects of future climate change on reptiles in general, and the Galápagos marine iguana in particular. Although it is unclear how climate change will affect the ENSO (Paltán et al., 2021), many studies predict that El Niño will become more frequent, and the majority of simulations considered to be most reliable predict imminent changes in its variability (Collins et al., 2010; Vecchi and Wittenberg, 2010). This may intensify the food shortages and lead to higher mortality rates in marine iguanas. Regardless of the ENSO, warming SSTs could cause the algae species eaten by iguanas to shift their range to deeper water (as is already observed in many species of marine organisms; Chaikin et al., 2022, 2024; Dulvy et al., 2008). If this occurs, iguanas will need to dive deeper or face a permanent reduction in access to food. Such a change would presumably be most harmful to the smaller individuals, which cannot dive as deep as larger iguanas (Vitousek et al., 2007; Wikelski, 2005) and are thus likely to experience the most drastic dietary restriction (Laurie and Brown, 1990a,b). Another possible result is a long-term decline in the overall body size of the species, as body size is a trade-off between nutritional and reproductive considerations in marine iguanas (Vitousek et al., 2007). This outcome seems especially likely, considering that decline in body size is a common response to climate change (Sheridan and Bickford, 2011), mostly in endotherms (Dubiner and Meiri, 2022; Ryding et al., 2021), but also in ectothermic vertebrates (Loehr et al., 2007; Moldowan et al., 2022).

The Galápagos marine iguana is an endemic, threatened species with several endangered and critically endangered subspecies (MacLeod et al., 2020). It is acutely exposed to the threats of climate change owing to its singular ecological and dietary specialization. Understanding the vulnerability of marine iguanas to ENSO is an increasingly pressing matter. If we are to conserve this unique species, it is important to understand how its physiology is adapted to cope with the natural climatic oscillations common to its native habitat, and how this vulnerability is further affected by anthropogenic ocean warming.

## Supplementary Material

10.1242/jexbio.250907_sup1 Supplementary information
