# Supplementary material for: Marine iguanas have lower metabolic rates during El Niño
Source: J Exp Biol. 2025 Sep 5;228(17):jeb250907. doi: 10.1242/jeb.250907 (PMC12450463; doi:10.1242/jeb.250907)
Supplement: Supplementary information [file jexbio-228-250907-s1.pdf]

Table S1. data for iguanas measured in this study. ENSO: El Nino Southern Oscillation (positive = El Nino); T_b: body temperature (°C); Mass: in g; SVL: snout-vent length (mm); TL: tail length, mm; girth: chest circumference (mm); HW & HL: head width & length (mm); RMR: O_2 consumption (ml/g/h).

Date	ENSO	Time	Location	Latitude	Longitude	ID	T_b	Mass	SVL	TL	Tail	Girth	HW	HL	Weather	RMR	Chip identifier
11/02/2024	positive	8:20	La Loberia	-0.923	-89.618	LL0a	32.6	3500	450	555	NA	NA	NA	NA	Sunny	NA	NA
11/02/2024	positive	8:25	La Loberia	-0.923	-89.618	LL0b	NA	3750	420	565	NA	NA	NA	NA	Sunny	NA	NA
11/02/2024	positive	8:30	La Loberia	-0.923	-89.618	LL0c	NA	2500	490	490	NA	NA	NA	NA	Sunny	NA	NA
11/02/2024	positive	8:35	La Loberia	-0.923	-89.618	LL00d	36.1	1470	310	441	NA	260	44	48	Sunny	0.094	985112007159938
11/02/2024	positive	10:00	La Loberia	-0.923	-89.618	LL01	36.3	3200	370	600	NA	370	5.6	70	Sunny	NA	NA
11/02/2024	positive	10:04	La Loberia	-0.923	-89.618	LL02	36.2	4700	425	613	NA	340	73	73	Sunny	NA	NA
11/02/2024	positive	10:17	La Loberia	-0.923	-89.618	LL03	34.4	1430	270	320	loss	280	54	54	Sunny	0.132	NA
11/02/2024	positive	10:24	La Loberia	-0.923	-89.618	LL04	35.1	3540	425	590	NA	375	67	67	Sunny	NA	900234000479364
11/02/2024	positive	10:29	La Loberia	-0.923	-89.618	LL05	36.7	3500	350	520	NA	225	64	59	Sunny	NA	NA
11/02/2024	positive	12:09	La Loberia	-0.923	-89.618	LL06	34.8	2400	318	501	NA	NA	NA	NA	Sunny	NA	NA
11/02/2024	positive	14:00	La Loberia	-0.923	-89.618	LL07	36.8	1330	280	430	NA	270	49	37	Sunny	0.217	900235000476358
11/02/2024	positive	12-14h	La Loberia	-0.923	-89.618	LL08	NA	2070	385	460	NA	280	47	58	Sunny	0.136	NA
11/02/2024	positive	12-14h	La Loberia	-0.923	-89.618	LL09	36.7	1860	350	560	NA	290	44	53	Sunny	NA	NA
11/02/2024	positive	12-14h	La Loberia	-0.923	-89.618	LL10	35.9	2469	399	405	NA	310	54	63	Sunny	NA	NA
11/02/2024	positive	12-14h	La Loberia	-0.923	-89.618	LL11	37.1	5700	480	450	loss	405	70	76	Sunny	NA	NA
11/02/2024	positive	15:30	La Loberia	-0.923	-89.618	LL12	37	1720	300	403	NA	298	46	52	Sunny	0.149	900235000479336
12/02/2024	positive	9:00	Punta Carola	-0.923	-89.618	PC01	NA	970	232	NA	NA	NA	NA	NA	Rainy	0.266	NA
12/02/2024	positive	11:00	Punta Carola	-0.923	-89.618	PC02	NA	1800	330	NA	NA	NA	NA	NA	Rainy	0.084	NA
12/02/2024	positive	13:00	Punta Carola	-0.923	-89.618	PC03	NA	1495	290	NA	NA	NA	NA	NA	Rainy	0.148	NA
12/02/2024	positive	15:00	Punta Carola	-0.923	-89.618	PC04	NA	1080	272	NA	NA	NA	NA	NA	Rainy	0.182	NA
12/02/2024	positive	17:00	Punta Carola	-0.923	-89.618	PC05	NA	2025	332	NA	bent	NA	NA	NA	Rainy	0.072	NA
13/02/2024	positive	9:08	Punta Carola	-0.896	-89.612	PC06	28.7	NA	NA	440	NA	NA	38	49	Partly cloudy	NA	NA
13/02/2024	positive	9:57	Punta Carola	-0.896	-89.612	PC07	30.4	1760	380	516	NA	270	47	55	Partly cloudy	0.101	900235000476861
13/02/2024	positive	10:03	Punta Carola	-0.896	-89.612	PC08	30	1230	310	NA	NA	250	40	48	Partly cloudy	NA	NA
13/02/2024	positive	10:07	Punta Carola	-0.896	-89.612	PC09	32.2	570	225	NA	NA	NA	NA	NA	Partly cloudy	0.625	NA
13/02/2024	positive	10:10	Punta Carola	-0.896	-89.612	PC10	32.5	NA	332	570	NA	325	50	56	Partly cloudy	NA	NA
13/02/2024	positive	10:23	Punta Carola	-0.896	-89.612	PC11	31.6	NA	350	570	NA	315	53	57	Partly cloudy	NA	NA
13/02/2024	positive	10:39	Punta Carola	-0.896	-89.612	PC12	NA	3620	440	600	NA	350	64	68	Partly cloudy	NA	NA
13/02/2024	positive	10:47	Punta Carola	-0.896	-89.612	PC13	NA	3300	470	630	NA	330	64	67	Partly cloudy	NA	900235000476943

13/02/2024	positive	11:53	Punta Carola	-0.896	-89.612	PC14	NA	1400	295	NA	NA	NA	NA	NA	Partly cloudy	0.13	900235000476854
13/02/2024	positive	13:30	Punta Carola	-0.896	-89.612	PC15	33.6	1675	335	390	NA	270	44	52	Partly cloudy	0.131	900235000476942
13/02/2024	positive	15:00	Punta Carola	-0.896	-89.612	PC16	36.2	1100	250	370	NA	230	39	46	Partly cloudy	0.159	NA
14/02/2024	positive	9:00	Playa Mann	-0.896	-89.609	PM01	NA	850	270	390	NA	260	NA	NA	Partly cloudy	0.221	900235000476923
14/02/2024	positive	11:00	Playa Mann	-0.896	-89.609	PM02	NA	415	213	309	NA	160	NA	NA	Partly cloudy	0.311	NA
14/02/2024	positive	13:00	Playa Mann	-0.896	-89.609	PM03	NA	960	230	390	NA	245	NA	NA	Partly cloudy	0.124	900235000476489
14/02/2024	positive	14:00	Playa Mann	-0.896	-89.609	PM04	NA	1270	325	NA	NA	NA	NA	NA	Partly cloudy	NA	NA
14/02/2024	positive	16:00	Playa Mann	-0.896	-89.609	PM05	NA	1470	325	NA	NA	NA	NA	NA	Partly cloudy	0.17	NA
11/03/2025	neutral	10:13	La Loberia	-0.923	-89.618	LL20	33	2380	335	450	NA	300	53	65	Sunny	0.104	NA
11/03/2025	neutral	11:53	La Loberia	-0.923	-89.618	LL21	32.4	1200	260	420	NA	270	41	48	Sunny	0.334	900235000486750
11/03/2025	neutral	14:30	La Loberia	-0.923	-89.618	LL22	27.5	975	260	440	NA	270	43	49	Rainy	0.307	NA
11/03/2025	neutral	15:30	La Loberia	-0.923	-89.618	LL23	30.7	1899	295	455	NA	280	41	50	Rainy	0.181	NA
12/03/2025	neutral	8:27	Punta Carola	-0.896	-89.612	PC17	32.7	2140	295	465	NA	320	46	53	Sunny	0.118	NA
12/03/2025	neutral	10:00	Punta Carola	-0.896	-89.612	PC18	28.4	4450	370	600	NA	370	63	61	Sunny	NA	NA
12/03/2025	neutral	11:30	Punta Carola	-0.896	-89.612	PC19	NA	2180	290	470	NA	365	46	53	Sunny	NA	NA
12/03/2025	neutral	11:40	Punta Carola	-0.896	-89.612	PC20	30.9	970	270	410	NA	360	39	47	Cloudy	0.191	NA
12/03/2025	neutral	13:00	Punta Carola	-0.896	-89.612	PC21	31.5	2890	345	590	NA	390	58	62	Rainy	0.104	NA
12/03/2025	neutral	14:50	Punta Carola	-0.896	-89.612	PC22	29.3	1755	295	455	NA	275	43	45	Rainy	0.125	NA
16/03/2025	neutral	8:30	La Loberia	-0.923	-89.618	LL24	28.8	2310	285	502	NA	312	54	57	Sunny	0.085	900235000107104
16/03/2025	neutral	9-10h	La Loberia	-0.923	-89.618	LL25	NA	NA	250	NA	NA	275	43	39	Sunny	NA	900235000903120
16/03/2025	neutral	9-10h	La Loberia	-0.923	-89.618	LL26	32.7	NA	NA	NA	NA	350	54	65	Sunny	NA	900235000476360
16/03/2025	neutral	10:00	La Loberia	-0.923	-89.618	LL27	30.4	2440	420	660	NA	480	69	64	Sunny	0.148	900235000903107
16/03/2025	neutral	10-11h	La Loberia	-0.923	-89.618	LL28	31.7	NA	360	519	NA	390	64	59	Sunny	NA	900235000907178
16/03/2025	neutral	10-11h	La Loberia	-0.923	-89.618	LL29	33	NA	370	564	NA	400	29	31	Sunny	NA	900235000477317
16/03/2025	neutral	10-11h	La Loberia	-0.923	-89.618	LL30	NA	NA	350	535	NA	NA	54	45	Sunny	NA	900235000903582
16/03/2025	neutral	10-11h	La Loberia	-0.923	-89.618	LL31	34.1	NA	320	480	NA	330	51	58	Sunny	NA	900235000907138
16/03/2025	neutral	11:30	La Loberia	-0.923	-89.618	LL32	32.2	1870	312	455	NA	NA	44	44	Sunny	0.143	900235000903084
16/03/2025	neutral	12-13h	La Loberia	-0.923	-89.618	LL33	35.1	NA	340	520	NA	330	54	65	Sunny	NA	900235000903104
16/03/2025	neutral	13:00	La Loberia	-0.923	-89.618	LL34	34.9	1530	335	465	NA	280	43	40	Sunny	0.196	900235000907176
16/03/2025	neutral	14:30	La Loberia	-0.923	-89.618	LL35	35	3100	390	455	NA	310	41	40	Sunny	0.109	900235000903091
16/03/2025	neutral	15-16h	La Loberia	-0.923	-89.618	LL36	38.2	NA	365	615	NA	370	65	75	Sunny	NA	900235000923009
16/03/2025	neutral	16:00	La Loberia	-0.923	-89.618	LL37	35.4	3290	380	570	NA	350	64	67	Sunny	0.113	900235000478488
17/03/2025	neutral	8:30	Punta Carola	-0.896	-89.612	PC23	33.8	1440	350	410	NA	420	46	40	Cloudy	0.15	NA

17/03/2025	neutral	8:30	Punta Carola	-0.896	-89.612	PC24	32	3110	380	585	NA	NA	NA	NA	Cloudy	NA	NA
17/03/2025	neutral	8:45	Punta Carola	-0.896	-89.612	PC25	28.9	4770	450	630	NA	NA	NA	NA	Cloudy	NA	NA
17/03/2025	neutral	8:50	Punta Carola	-0.896	-89.612	PC26	32.4	3430	380	520	NA	NA	NA	NA	Cloudy	NA	NA
17/03/2025	neutral	9:00	Punta Carola	-0.896	-89.612	PC27	33.2	1250	280	450	NA	265	45	38	Cloudy	0.188	900235000917782
17/03/2025	neutral	9:25	Punta Carola	-0.896	-89.612	PC28	31.8	2710	400	505	NA	NA	NA	NA	Cloudy	NA	NA
17/03/2025	neutral	9:29	Punta Carola	-0.896	-89.612	PC29	31.7	3790	450	560	NA	NA	NA	NA	Cloudy	NA	NA
17/03/2025	neutral	9:54	Punta Carola	-0.896	-89.612	PC30	28	1750	290	470	NA	NA	NA	NA	Cloudy	NA	NA
17/03/2025	neutral	10:00	Punta Carola	-0.896	-89.612	PC31	29.3	1290	285	260	NA	245	49	43	Cloudy	0.14	NA
17/03/2025	neutral	10:30	Punta Carola	-0.896	-89.612	PC32	30.3	1800	330	495	NA	NA	NA	NA	Cloudy	NA	NA
17/03/2025	neutral	10:39	Punta Carola	-0.896	-89.612	PC33	31.3	4180	415	568	NA	NA	NA	NA	Cloudy	NA	NA
17/03/2025	neutral	10:50	Punta Carola	-0.896	-89.612	PC34	29.4	1590	280	415	NA	NA	NA	NA	Cloudy	NA	NA
17/03/2025	neutral	10:50	Punta Carola	-0.896	-89.612	PC35	30.1	2180	290	495	NA	285	NA	NA	Cloudy	NA	NA
17/03/2025	neutral	12:30	Punta Carola	-0.896	-89.612	PC36	35	1700	310	460	NA	NA	55	47	Cloudy	0.125	NA
17/03/2025	neutral	13:35	Punta Carola	-0.896	-89.612	PC37	36.3	975	265	420	NA	230	41	35	Cloudy	0.253	NA
18/03/2025	neutral	8:06	La Loberia	-0.923	-89.618	LL38	27.3	2380	365	555	NA	305	48	58	Cloudy	NA	NA
18/03/2025	neutral	8:24	La Loberia	-0.923	-89.618	LL39	26.7	3750	418	600	NA	335	58	70	Cloudy	NA	NA
18/03/2025	neutral	8:46	La Loberia	-0.923	-89.618	LL40	26.4	2880	390	540	NA	305	57	65	Cloudy	NA	900235000922044
18/03/2025	neutral	9:08	La Loberia	-0.923	-89.618	LL41	26.7	1640	286	426	NA	266	41	47	Cloudy	NA	900235000906346
18/03/2025	neutral	9:10	La Loberia	-0.923	-89.618	LL42	26.5	1150	295	420	NA	246	42	45	Cloudy	NA	900235000906340
19/03/2025	neutral	9:35	La Loberia	-0.923	-89.618	LL43	27.6	5800	510	702	NA	409	65	73	Cloudy	NA	NA
18/03/2025	neutral	9:40	La Loberia	-0.923	-89.618	LL44	27.3	1950	352	480	NA	280	NA	NA	Cloudy	NA	900235000922038
18/03/2025	neutral	9:58	La Loberia	-0.923	-89.618	LL45	28.5	4150	412	607	NA	389	56	69	Cloudy	NA	900235000906336
18/03/2025	neutral	10:17	La Loberia	-0.923	-89.618	LL46	27.6	2150	290	538	NA	335	42	59	Cloudy	NA	900235000906345
18/03/2025	neutral	10:24	La Loberia	-0.923	-89.618	LL47	29.8	5640	487	728	NA	398	61	68	Cloudy	NA	900235000906134
18/03/2025	neutral	10:40	La Loberia	-0.923	-89.618	LL48	28.5	5900	468	401	NA	431	67	78	Cloudy	NA	900235000922037

Journal of Experimental Biology • Supplementary information
